# Management of Pain and Cachexia in Pancreatic Cancer: Protocol for Two Systematic Reviews, Network Meta-Analysis, Surveys, and Focus Groups

**DOI:** 10.2196/46335

**Published:** 2023-04-04

**Authors:** Danielle Amanda Roberts, Eila Watson, Christopher Macdonald, Yarunnessa Khan, Sarah Prideaux, Alwin Puthiyakunnel Saji, Emilia Postaleniec, Jashan Selvakumar, Mahta Haghighat Ghahfarokhi, Brian Davidson, Kurinchi Gurusamy

**Affiliations:** 1 Division of Surgery and Interventional Science Hampstead Campus University College London London United Kingdom; 2 Supportive Cancer Care Research Group Faculty of Health and Life Sciences Oxford Brookes University Oxford United Kingdom; 3 Pancreatic Cancer UK London United Kingdom; 4 Patient and Public Involvement Representative England United Kingdom; 5 School of Medicine Cardiff University Cardiff United Kingdom; 6 Leicester Medical School The University of Leicester Leicester United Kingdom; 7 St George's, University of London Medical School London United Kingdom; 8 Guy’s, King’s and St Thomas’ School of Medical Education Kings College London London United Kingdom

**Keywords:** cachexia, pain relief, palliative care, pancreatic cancer, quality of life, systematic review

## Abstract

**Background:**

Approximately 75% of people with pancreatic cancer experience pain, and >50% of them have cachexia (weakness and wasting of the body). However, there is considerable uncertainty regarding the management of these distressing symptoms.

**Objective:**

Our primary objectives are to compare the relative benefits and harms of different interventions for pain in people with unresectable pancreatic cancer and for prevention and treatment of cachexia due to pancreatic cancer, through systematic reviews and network meta-analysis. Our secondary objectives are to develop an evidence-based clinical care pathway to manage pain and prevent and treat cachexia in people with pancreatic cancer through surveys and focus groups involving patients, carers, and health care professionals.

**Methods:**

We will perform 2 systematic reviews of the literature related to pain and cachexia in people with pancreatic cancer using searches from Cochrane Library, MEDLINE, Embase, Science Citation Index, and trial registries. Two researchers will independently screen for eligibility and identify randomized controlled trials (no language or publication status restriction), comparing interventions for pain or cachexia based on full-texts for articles shortlisted during screening. We will assess risk of bias in the trials using the Cochrane risk of bias tool (version 2.0) and obtain data related to baseline prognostic characteristics, potential effect modifiers and outcome data related to overall survival, health-related quality of life, treatment-related complications, and resource utilisation. We aim to conduct network meta-analysis on outcomes with multiple treatment comparisons where possible, otherwise, meta-analysis with direct comparisons, or narrative synthesis. We will perform various subgroup and sensitivity analyses. Using information obtained from both systematic reviews, we will conduct 2 surveys: one directed to patients or carers to assess acceptability of interventions, and the other to health care professionals to assess feasibility of delivery in the National Health Service. Four mixed focus groups will be conducted to evaluate findings and foster consensus in the development of the care pathway.

**Results:**

Funding was awarded from April 2022 (NIHR202727). Both systematic review protocols were prospectively registered on PROSPERO in May 2022. Formal searches began thereafter. Approval by the University College London Research Ethics Committee (23563/001) was received in December 2022. Data collection began in January 2023; data analysis will begin in May 2023 (completion expected by October 2023).

**Conclusions:**

This study will comprehensively encompass major interventions for management of pain in people with unresectable pancreatic cancer, and prevention and treatment of cachexia in people with pancreatic cancer. Key stakeholders will facilitate the development of an evidence-based care pathway, ensuring both acceptability and feasibility. The project ends in April 2024 and published results are expected within 12 months of completion. We aim to present the findings through patient group websites, conferences, and publications, irrespective of the findings, in a peer-reviewed journal.

**International Registered Report Identifier (IRRID):**

DERR1-10.2196/46335

## Introduction

### What Is the Problem Being Addressed?

In the United Kingdom, 11,700 people were diagnosed and 10,000 people died from pancreatic cancer in 2020 [1]. The incidence of pancreatic cancer is increasing in the United Kingdom and in many other countries worldwide [2-9]. If the trend continues, it is likely to become the third-most common cause of cancer death in the United Kingdom by 2030 [10]. Surgical resection remains the only treatment with the potential for long-term survival and cure. However, only about 10% to 20% (n=~61,203) of patients are suitable for resection [8,11-13]. In people who do not undergo resection, the main options are chemotherapy, chemoradiotherapy, and treatment of symptoms [13-18]. The major symptoms related to pancreatic cancer, particularly in those with unresectable cancers, are pain, cachexia, and jaundice. In this work, we will be looking at the evidence for the management of pain and cachexia.

### Pain

Pancreatic cancer can cause severe pain. It is estimated that 70% to 80% of people with pancreatic cancer seek medical help for the treatment of abdominal or back pain [19,20]. The 2 major mechanisms of pain are pancreatic ductal obstruction and pancreatic neuropathy (increased activation of pain nerve fibers) [20]. The main treatment options evaluated for pancreatic cancer pain include nonopioid analgesics such as nonsteroidal anti-inflammatory drugs (NSAIDs), opioid analgesics such as morphine, corticosteroids, neuropathic pain medications such as gabapentin or pregabalin, palliative radiotherapy, thoracoscopic splanchnicectomy (severing the splanchnic nerves), celiac plexus nerve blocks or neurolysis, and other treatments include pancreatic duct stenting, acupuncture, and hypnosis [20-24]. Review 1 is a systematic review focusing on the benefits and harms of the different pain control strategies in people with unresectable pancreatic cancer. There are no previous network meta-analyses (NMA) on the topic. Head-to-head comparison systematic reviews included comparison of celiac plexus nerve block plus analgesics with analgesics alone [23,25,26] and videothoracoscopic splanchnicectomy [27], stereotactic radiotherapy [28], various ablative therapies [29], kanglaite (Chinese medicine) in addition to chemoradiotherapy versus chemoradiotherapy alone [30]. The current clinical guidelines on the management of pain in pancreatic cancer are based on the World Health Organization’s 3-step ladder for cancer pain [31]. The National Institute of Health and Care Excellence (NICE) suggests nonpharmacological treatments such as coeliac plexus blocks when the pharmacological treatment fails or is unacceptable because of adverse events [32], a recommendation that has been questioned [21]. The NICE acknowledged the uncertainty in the recommendation [32].

### Cachexia

Cancer cachexia is a multifactorial syndrome defined by an ongoing loss of skeletal muscle mass (with or without loss of fat mass) that cannot be fully reversed by conventional nutritional support and leads to progressive functional impairment [33]. An estimated 55% to 85% (n=~170) of people with pancreatic cancer have cachexia [34,35]. The major proposed mechanism for cachexia of pancreatic cancer is decreased appetite, decreased production and increased lysis of lipids, increased protein lysis, decreased liver function, fat malabsorption, and decreased muscle mass due to increased cytokines and tumor-derived factors [34,36,37]. Mechanical intestinal obstruction due to the cancer, pancreatic exocrine insufficiency, and nutritional deficiency because of the combination of the above factors are other potential mechanisms [34,36,37]. Interventions evaluated for the prevention and treatment of cachexia include pancreatic enzyme replacements, increased calorie intake, ketogenic diet, amino acid supplementation, nutritional supplements, appetite stimulants, corticosteroids, NSAIDs, progesterone analogues such as megestrol acetate, cytokine inhibitors such as melatonin, omega-3 fatty acids, and exercises to improve muscle mass [37-39]. Review 2 is a systematic review of the prevention and treatment of cachexia in people with pancreatic cancer. There are no previous NMAs on the topic. The existing systematic reviews on the topic are different from this study because of the types of studies included [40,41], types of interventions included [40-42], risk of bias assessment [40-42], and the type of outcome measures studied [40-42]. The NICE guideline on pancreatic cancer does not recommend anything specific for cachexia [32]; however, only nutritional interventions (for cachexia) were evaluated.

### Why Is This Research Important to Patients and Health and Care Services?

Increased pain decreases the health-related quality of life [43]. Cancer cachexia can lead to decreased health-related quality of life and may increase treatment complications, health care costs, and deaths [44]. Low performance status due to cachexia may preclude patients receiving more toxic but effective treatments such as the combination chemotherapy FOLFIRINOX [45]. The research questions included in this study are among the top 10 research priorities of the James-Lind Alliance Priority Setting Partnership on pancreatic cancer therapy involving people with pancreatic cancer in Germany, their carers, and clinicians treating them [46,47], that is, “How can the best treatment for each individual patient with pancreatic cancer be identified?” and “Does nutrition influence the survival or quality of life of patients with pancreatic cancer?” Furthermore, the use of evidence-based clinical care pathways in the National Health Service (NHS) helps to streamline care and avoid health care inequities and therefore would be beneficial for the management of pain and cachexia in people with pancreatic cancer.

### Aims and Objectives

The overarching aim of this study is to answer the following research question: “What is the best treatment and NHS care pathway to decrease pain and cachexia and improve health-related quality of life in people with pancreatic cancer?”

The primary objectives include the following:

- To compare the benefits and harms of different treatments for pancreatic cancer pain through network meta-analyses, and to generate rankings of the different treatments according to their safety and efficacy.
- To compare the benefits and harms of different interventions in the prevention and treatment of pancreatic cancer cachexia through a systematic review (and meta-analysis when possible).

The secondary objectives include the following:

- To identify the gaps in the existing research that cause uncertainty in decision-making (based on the systematic review and NMA).
- To develop an evidence-based pathway for the management of pain and for the prevention and treatment of cachexia (using evidence collected as part of primary objectives), through surveys and focus group discussions with patients, carers, and NHS clinicians.

## Methods

The bulk of the study is contained within work package 1, in which we will complete 2 comprehensive systematic reviews and meta-analyses on the management of pain and cachexia in pancreatic cancer. Work package 2 forms a smaller part of the overall study, yet it will involve surveys and focus groups of patients, carers, and health care professionals, leading to the development of a proposed evidence-based clinical care pathway for the management of pain and cachexia in people with pancreatic cancer.

### Work Package 1: Systematic Reviews and NMA

We will perform two systematic reviews:

- Management of pain in people with unresectable pancreatic cancer: a systematic review of randomized controlled trials and NMA (review 1).
- Prevention and treatment of cachexia in people with pancreatic cancer: a systematic review of randomized controlled trials (review 2).

We will register the protocols in PROSPERO (International Prospective Register of Systematic Reviews) and conduct and report the systematic review according to the PRISMA (Preferred Reporting Items for Systematic Reviews and Meta-Analyses) statement and its extension for NMA [48,49] (Table 1).

**Table 1:** Eligibility criteria for systematic reviews.

	Review 1	Review 2
Title	Management of pain in people with unresectable pancreatic cancer	Prevention and treatment of cachexia in people with pancreatic cancer
Type of studies	All randomized controlled trials regardless of the publication status, year of publication, and language of publication	All randomized controlled trials regardless of the publication status, year of publication, and language of publication
Setting	Primary, secondary, tertiary, or community care	Primary, secondary, tertiary, or community care
Types of participants	People with unresectable pancreatic cancer	People with pancreatic cancer regardless of whether the tumor was resectable or not
**Types of interventions**		
	NSAIDs,^a^ opioid analgesics, neuropathic pain medications, thoracoscopic splanchnicectomy, celiac plexus blocks or neurolysis, splanchnic nerve blocks or neurolysis, radiotherapy, pancreatic duct stenting, acupuncture, and hypnosis	Pancreatic enzyme replacements, increased calorie intake, ketogenic diet, amino acid supplementation, nutritional supplements (oral or parenteral), appetite stimulants, corticosteroids, NSAIDs, progesterone analogues, cytokine inhibitors, omega-3 fatty acids, and exercises to improve muscle mass
	Within each review, the above interventions will be considered if used either alone or in combination with other interventions listed and compared with another intervention (or combination of interventions) listed above.	Within each review, the above interventions will be considered if used either alone or in combination with other interventions listed and compared with another intervention (or combination of interventions) listed above.

^a^NSAIDs: nonsteroidal anti-inflammatory drugs.

### Outcomes

The choice of outcomes is based on the Core Set of Patient-Reported Outcomes in Pancreatic Cancer (ie COPRAC) study that involved patients with pancreatic cancer and health care professionals involved in their care [50]. We will collect all outcomes at 3 time points: until 3 months from randomization or intervention, 3 to 12 months from randomization or intervention, and beyond 12 months.

The primary outcomes include the following:

1. Overall health-related quality of life (any validated scale)
2. Pain (however defined by authors) (this will be secondary outcome for review 2)
3. Cachexia (however defined by authors) (this will be secondary outcome for review 1)

The secondary outcomes include the following:

1. Consumption of analgesics (drug, quantity, and frequency) (review 1 only)
2. Change in body weight (total amount and percentage of body weight) (review 2 only)
3. Death from any cause (all-cause mortality)
4. General health (any validated scale)
5. Physical ability (any validated scale)
6. Ability to work and do usual activities (however defined by authors)
7. Satisfaction with services and care organization (any validated scale)
8. Relationship with partner and family (any validated scale)
9. Serious adverse events (ICH-GCP [51] or any other definitions used by authors)
10. Number of hospital attendances and hospital admissions
11. The total length of hospital stays (planned admissions related to all components of treatments and admissions related to observation or treatment of complications)

### Search Strategy

The following electronic databases will be searched to retrieve all eligible studies: The Cochrane Central Register of Controlled Trials (CENTRAL) in the Cochrane Library; MEDLINE (OvidSP); Embase (OvidSP); and Science Citation Index Expanded (Web of Knowledge); Conference Proceedings, from inception to date of search using free text and controlled vocabulary terms; ClinicalTrials.gov and the World Health Organization International Clinical Trials Registry Platform [52], which searches various trial registers, including ISRCTN and ClinicalTrials.gov. The search strategies are provided on the web-based published study registration records on PROSPERO (CRD42022333239 and CRD42022333241).

Additionally, we will search the references of the identified trials and the existing recent major systematic reviews on the topics to identify additional trials for inclusion. We will also contact the study authors to identify further trials and obtain aggregate data from unpublished studies.

### Data Collection and Management

#### Selection of Studies

Two review authors will independently identify trials for inclusion by screening the titles and abstracts and make the final selection for inclusion based on the full-text articles (after translation if required) selected during screening. We will resolve any discrepancies through discussion and arbitration. The process will be documented to enable the completion of the PRISMA flowchart.

#### Data Collection

Two review authors will independently extract the following data using a prepiloted data extraction form:

1. Intervention and control details (including who delivers it, dose, how long and how frequently it is delivered, and compliance as appropriate)
2. Outcome definition, scale, and data
3. Data on potential effect modifiers: both reviews: age, performance status; review 2: presence of pancreatic exocrine insufficiency; resectable versus unresectable pancreatic cancer
4. Length of follow-up
5. Information to assess risk of bias
6. Other data include year and language of publication, country and setting, ethnicity of participants, recruitment period, and inclusion and exclusion criteria.

#### Assessment of Bias

We will use the Cochrane risk of bias tool version 2.0 to assess the risk of bias in the included randomized controlled trials [53]. We will assess reporting bias by the completeness of search.

### Meta-analysis of Clinical Effectiveness

#### Measures of Treatment Effect

For binary outcomes, we will calculate the odds ratio with 95% credible interval (CrI). For continuous outcomes, we will calculate the mean difference (if trials used same scale) or standardized mean difference (if trials used different scales) with 95% CrI. For count outcomes, we will calculate the rate ratio with 95% CrI. For time-to-event data, we will calculate hazard ratio with 95% CrI.

#### Data Synthesis

We will conduct NMA on all outcomes with multiple treatment comparisons wherever possible. We will obtain a network plot to understand the network geometry and ensure that the trials are connected by interventions. We will report only the direct pairwise meta-analysis for comparisons that are not connected to the network. We will summarize the population and methodological characteristics of the trials included in the NMA in a table based on pairwise comparisons and ensure that the transitivity assumption is reasonable. If there are concerns about the transitivity assumption, we will perform only direct comparisons. We will conduct a Bayesian NMA by using the Markov chain Monte Carlo method in OpenBUGS 3.2.3 as per the guidance from the NICE Decision Support Unit (NICE DSU) documents using study-level data and appropriate likelihood, and link functions [54]. We will calculate the additive main effects, 2-way interaction, and the full-interaction models [55] to capture the effect of a component, interactions between the components and the overall effect of a combination of components. We will use the model fit to guide the selection of the model to be reported, when we perform a component NMA. We will use the standard of care as the reference group.

We will perform a fixed-effect model and random-effects model for the NMA, and report the more conservative model. The codes that we use for the analysis will account for the correlation between the effect sizes from the studies with more than 2 groups [54]. We will use a hierarchical Bayesian model using “vague” priors and 3 different sets of initial values (to ensure convergence of values), using the codes provided by NICE DSU. We will estimate the probability that each intervention ranks at one of the possible positions by using the NICE DSU codes. We will obtain the surface under the cumulative ranking curve (cumulative probability) and rankogram [56,57].

#### Presentation of Results

We will present the effect estimates with 95% CrI for each pairwise comparison calculated from the direct comparisons and NMA. We will also present rankograms and surface under the cumulative ranking curve [58,59]. We will present “Summary of Findings” tables for each of the primary and secondary outcomes using the methods described by the GRADE Working Group for presenting the Summary of Findings for NMA [60].

#### Dealing With Missing Data

In the first instance, we will endeavor to contact trial investigators to obtain missing data. We will perform an intention-to-treat analysis, whenever possible [61]; otherwise, we will use the data available to us. We will conduct best-worst case and worst-best case scenario analyses as sensitivity analyses for binary outcomes, whenever possible. For continuous outcomes, we will impute the mean and SD from median and *P* values according to the guidance in the Cochrane Handbook if the data seem to be normally distributed [62].

#### Alternatives to Meta-analysis

For outcomes where meta-analysis is not possible (eg, where the data were not normally distributed), we will present the studies in a table and report the median and quartiles of the differences. When it is inappropriate to perform a meta-analysis because of major differences in the types of participants included in the studies, we will summarize the information in a table and perform only a narrative synthesis. For the narrative synthesis, we will present the results systematically by the comparisons for each outcome and will report the findings according to “Synthesis Without Meta-analysis” guidelines [63].

### Assessment and Investigation of Heterogeneity and Inconsistency

#### Heterogeneity

We will assess clinical and methodological heterogeneity by carefully examining the characteristics and design of the included trials. The potential sources of clinical heterogeneity include age, performance status, presence of pancreatic exocrine insufficiency; resectable versus unresectable pancreatic cancer, baseline risk (may be related to when the trial was conducted reflecting improvement in supportive care), variations in the intervention, different definitions used for outcomes, different scales used for assessment of outcomes, and period of follow-up.

We may encounter a diverse range of outcome measures, that is, for pain and health-related quality of life, using different scales. To account for this we will convert the available data into standardized mean difference and the SE that can be directly used in the NMA applying the standard WinBUGS codes available from the NICE DSU documents [54], that is, we will use the codes for combining “treatment differences.” We may also encounter cases in which pain may be reported as “pain response” in some trials, but reported as continuous outcomes in other trials. In these cases, we will convert the available data into standardized mean difference using the methods provided in the Cochrane Handbook (version 6.3, Section 10.6) [62] for the conversion of odds ratio to standardized mean difference (and its SE). Once converted into standardized mean difference, this can be used in the same way as above in the NMA. We will consider this variation in the way that outcomes are reported as a source of heterogeneity and address this by meta-regression and subgroup analysis, as appropriate.

#### Investigation of Heterogeneity

We will assess statistical heterogeneity by comparing the results of the fixed-effect model meta-analysis and the random-effects model meta-analysis, between-study variance, and by calculating NMA-specific *I*^2^ [64]. If we identify substantial, clinical, methodological, or statistical heterogeneity, we will explore and address the heterogeneity in subgroup analysis and meta-regression by using the methods and codes described in the NICE DSU documents [65].

#### Inconsistency

We will evaluate the plausibility of transitivity assumption by looking at the inclusion and exclusion criteria in the studies and limit the NMA to a subset of trials, where transitivity assumption is reasonable.

We will assess inconsistency (statistical evidence of the violation of transitivity assumption) by fitting both an inconsistency model and a consistency model. We will use the inconsistency models employed in the NICE DSU manual [59], as we will use a common between-study SD. In addition, we will use the design-by-treatment full interaction model and inconsistency factor plots to assess inconsistency [56,66]. If there was evidence of inconsistency, we will identify areas in the network where substantial inconsistency might be present in terms of clinical and methodological diversities between trials and, when appropriate, limit NMA to a more compatible subset of trials.

#### Sensitivity Analysis

In addition to the best–worst case scenario and worst–best case scenario sensitivity analyses mentioned above, we will also perform a sensitivity analysis excluding the trials in which mean or SD or both were imputed and use different imputed SDs.

### Work Package 2: Development of the Clinical Care Pathway

The information from work package 1 (both systematic reviews and NMA) will be supplemented by surveys and focus group discussions involving patients, carers, and health care professionals. We aim to engage these key stakeholders in a discussion about the acceptability and feasibility of interventions toward developing an evidence-based clinical care pathway for the management of pain and cachexia in pancreatic cancer.

### Surveys

Two web-based surveys will be performed: one directed to patients or carers and the other to health care professionals. Both surveys will address pain and cachexia symptoms in turn and will precede the focus group discussions. Both surveys will include a question asking respondents whether they would be willing to participate in a focus group to further discuss the acceptability and feasibility of the different treatments toward the development of a care pathway for pancreatic cancer pain and cachexia. In this way, we will recruit members for the subsequent focus group discussions. The surveys will be hosted via a web-based survey platform using REDCap software. We will also offer a word-processing version, as well as paper surveys with stamped return envelopes, to increase participation. There will be an appropriate mix of closed questions to allow for quantitative analysis and open questions enabling free-text, qualitative responses. The survey templates are available from the researchers upon request. We will use the Checklist for Reporting Results of Internet E-Surveys (CHERRIES) when formally reporting the results of the surveys for publication [67].

#### First Survey

For the sample frame, people with pancreatic cancer and their carers are identified through the Pancreatic Cancer UK patient network.

We expect approximately 50 responses for this survey. Although we recognize the importance of gaining views directly from people with pancreatic cancer, we have included carers due to the nature of this severe illness meaning people with pancreatic cancer and symptoms of the pan and cachexia may be too unwell to answer for themselves, and thus carers could provide valuable insights and views on behalf of people with pancreatic cancer.

The first survey aims to direct the focus group discussions toward deciding on the acceptability of the interventions (identified from the systematic reviews). We will provide the details of the procedure (what the procedure involves in plain language), and the effectiveness and complications of the procedures (again in plain language). Patients and carers will be asked to rank the treatments in the order of their preference or acceptability, according to how acceptable or tolerable they consider the treatment to be. We will summarize the acceptability (after stratification by symptom) in bar charts and pie charts.

#### Second Survey

For the sampling frame, health care professionals in NHS are identified through the Pancreatic Cancer UK clinical network.

We will also use the National Cancer Research Institute Upper Gastrointestinal Group (Pancreatic cancer Workstream) to publicize and increase the outreach of the survey. We expect around 50 responses for this web-based survey.

The purpose of the second survey will be to help inform the focus group discussions to understand the feasibility of performing the above interventions (identified in the systematic reviews) in the NHS and where best to deliver the intervention (ie, community, primary care, secondary care, specialist centers, hospice, etc). Health care professionals will be asked to score each intervention on a scale of 1 to 10, according to how feasible or realistic they consider for it to be delivered in NHS. We will summarize the feasibility of the treatments (after stratification by the category of the health care professional) in bar charts and pie charts.

### Focus Group Discussions

We will conduct focus group discussions involving patients, their carers, and health care professionals to develop a care pathway using the information from the systematic reviews (work package 1) and the surveys described above. Having a rich, mixed focus group (50% patients and carers and 50% health care professionals) will enable all participants to discuss their respective opinions and comments regarding the acceptability and feasibility of treatments, thereby enabling a consensus to be reached.

We will present the evidence from the systematic reviews and meta-analysis, and acceptability of treatments for each of pain and cachexia in two ways:

- ordered by the number of people who ranked an intervention as the most acceptable treatment
- ordered by their weighted ranking score using weights applied in reverse, for example, if there were 5 interventions, the most acceptable intervention will get a weight of 5, the second most acceptable intervention a weight of 4, the third most acceptable intervention a weight of 3, and so on.

Similarly, we will then present the feasibility of treatments in the NHS for each of pain and cachexia in 2 ways:

- ordered by the number of people who ranked an intervention as the most feasible treatment
- ordered by the average feasibility score.

After the presentation of the results of the surveys, the focus group will begin in-depth discussions to review the findings. The focus group discussions will focus around areas of contention—that is, interventions that are acceptable to patients but not very feasible to deliver in NHS or vice versa, or interventions that are highly acceptable but limited efficacy is found in the systematic review. Additionally, further discussions will take place regarding the most appropriate site of delivery of interventions. The templates for the focus group discussions are available from the researchers on request. We anticipate 2 focus group discussions (involving 8 to 12 participants each) covering each symptom (ie, 2 focus groups for pain interventions and 2 focus groups for cachexia interventions). Each round will be iterative, in that findings from the first focus groups in each symptomology will feed into each of the second focus group discussions. Each focus group’s meeting is expected to last from 60 to 90 minutes. We plan to conduct the focus group meetings in-person; however, we have the flexibility to conduct the meetings on the web via Microsoft Teams or using a hybrid model, if preferable for participants. We will record all focus group discussions and use General Data Protection Regulation–compliant transcription services for transcribing the discussions for analysis. The transcript will not contain any identifiable participant details.

The focus group discussions will be analyzed using the Framework method [68], using a preliminary logic model developed following Phase 1 of the study to inform the development of the analytical framework. This logic model will describe the theory underlying a potential care pathway for the management of pain and cachexia and will use a Situation (*pain, cachexia*)–Inputs (*resources*)–Outputs (*activities*)–Mechanism –Outcomes (*short and longer terms*) configuration [69,70]. The focus group data will be coded and mapped onto this framework and further themes and subthemes will be developed and used to modify the logic model as required. Regular research team discussions will be held to ensure that the coding scheme reflects the data. We will use the Standards for Reporting Qualitative Research when formally reporting the results of the focus group discussions for publication [71].

We anticipate the development of a proposed care pathway in further iterative communications among the research team and relevant additional health care professionals (depending upon the intervention) identified through National Cancer Research Institute Upper Gastrointestinal Group (Pancreatic cancer Workstream), thus reaching a final agreement via email or teleconferences.

### Patient and Public Involvement

The research team includes a patient representative who was involved in the preparation of the grant project proposal and determined that this research was important to patients. The patient representative will be part of the research oversight committee for the project and will help to facilitate the focus group discussions.

### Ethics Approval

This study was approved by the University College London Research Ethics Committee (Ethics number: 23563/001).

## Results

The funding for this study has been awarded from April 2022 to April 2024. The actual project start date occurred on schedule in April 2022. Immediately, prior to any study selection or formal searches, both systematic review protocols were prospectively registered publicly on the PROSPERO database in May 2022, along with the formal search strategies (CRD42022333239 and CRD42022333241). Formal searches and subsequent study screening began shortly thereafter. Data collection began in January 2023. Ethical approval (with regard to the surveys and focus groups pertaining to the second part of the project) was received in December 2022, and hence the full project protocol was prepared for publication in January 2023. Data collection began in January 2023 and data analysis is due to begin in May 2023 (expected to be completed by October 2023). Surveys and focus groups will run thereafter. The project ends in April 2024 and results are expected to be published within 12 months of project completion.

## Discussion

### Strengths and Limitations of This Study

This study will provide comprehensive coverage of all the major interventions for the management of pain in people with pancreatic cancer not suitable for pancreatic resection, and for the prevention and treatment of cachexia in people with pancreatic cancer regardless of whether they are suitable for pancreatic resection. It will also be the first time for network meta-analysis to be used for the systematic review on the management of pain in people with unresectable pancreatic cancer, which has several advantages in terms of evaluating multiple treatment comparisons, and enabling calculation of additive main effects, 2-way interactions, and the full interaction models, which allow us to find out if there are any interactions between different combinations of treatments. However, the main limitation of the study is that sufficient outcome data may not be available from the randomized trials to enable network meta-analysis to be performed.

Nevertheless, this study will identify the gaps in the existing research that cause uncertainty in decision-making in the management of pain and cachexia in people with pancreatic cancer. A further strength is that the study will consult key stakeholders in the development of an evidence-based care pathway for pain and cachexia in pancreatic cancer, ensuring acceptability for patients and carers and feasibility for delivery in the NHS.

### Dissemination

The team will disseminate the findings of the research through patients group websites, social media groups, conferences, and publish the findings in peer-reviewed journals.

## Appendix

Multimedia Appendix 1 Peer review reports.

## Abbreviations

CHERRIES: Checklist for Reporting Results of Internet E-Surveys
CrI: credible interval
NHS: National Health Service
NICE DSU: National Institute of Health and Care Excellence Decision Support Unit
NMA: network meta-analysis
NSAIDs: nonsteroidal anti-inflammatory drugs
PRISMA: Preferred Reporting Items for Systematic Reviews and Meta-Analyses
PROSPERO: International Prospective Register of Systematic Reviews

## Data Availability

For the systematic reviews in work package 1, all the data will be available from the published report. Regarding data generated from the surveys and focus group discussions in work package 2, we will provide summary data only, in line with the data management plan that was ethically approved.
