# Supplementary material for: Management of Pain and Cachexia in Pancreatic Cancer: Protocol for Two Systematic Reviews, Network Meta-Analysis, Surveys, and Focus Groups
Source: JMIR Res Protoc. 2023 Apr 4;12:e46335. doi: 10.2196/46335 (PMC10139686; doi:10.2196/46335)
Supplement: Multimedia Appendix 1 [file resprot_v12i1e46335_app1.pdf]

NIHR | National Institute for Health Research

Research for Patient Benefit Review Summary

Reference Number	NIHR202727
Research Title	Management of pain and cachexia in pancreatic cancer: Systematic reviews of randomised controlled trials and network meta-analysis
Chief Investigator	Dr Danielle Roberts
Contracting Organisation	University College London Hospitals NHS Foundation Trust
Total Amount Requested	£148,724.00
Reviewer Number	1

Table Of Contents

1. Reviewer Expertise
2. Relevance of the proposed research
3. Quality of the proposed work
4. Strength of the research team
5. Impact of the proposed work
6. Value for money
7. Involvement of patients and the public
8. Additional Comments

1. Reviewer Expertise

**Please indicate the nature of your expertise by clicking on the appropriate tick box(es) below:**
Clinician in the same/a very similar field, Researcher in a broadly related field

If the tick boxes above do not adequately capture the nature of your expertise, please briefly provide details in the box below (or use it to give us more detail about your expertise if you wish):

2. Relevance of the proposed research

i) How relevant and important is the proposed research to the priorities and needs of the NHS, and does it offer a health/healthcare solution with demonstrable benefit to patients?<br>ii) Does the application demonstrate an awareness and understanding of previous relevant research or developments in this area?<br>iii) To what extent does the proposed research add distinct value or advance existing knowledge in this area, taking into account wider ongoing or completed research?
i. highly relevant; an unmet need for these patients. Most patients have unresectable disease, or if resected recur. Pain and cachexia are highly prevalent. Synthesis of data is needed<br><br>ii. yes, there appears to be a suitable number of studies for SR and MA<br><br>iii. This is a topic area in need of review. This work, and through collaboration with pancreatic cancer UK, will raise the profile of this area and need for treatment. I suspect there will be useful areas shown where there can be improvement in outcomes from the generated data and for further areas for study

3. Quality of the proposed work

**Research Design**<br>i) How appropriate is the research design in relation to the stated objectives?<br>ii) To what extent is the proposed design and methodology for all elements of the research well defined, appropriate, valid and feasible within the timeframe and resources requested?<br>iii) What are the strengths and weaknesses of the research design as proposed?<br>iv) To what extent does the research show originality and innovation?
i. I have no issues with the proposed SR and network MA. I am not an expert in this field but the methods are standard<br><br>I have issue with the WP2. The team propose to use online surveys to obtain views from patients/carers. This method will have a low 'hit rate' and those that respond are likely to have an experience, or some other factor, that makes them more likely to respond. This may mean that the data that is generated is not wholly generalisable. Whilst I have no objection to this method I would suggest that it is augmented by a dissemination of q'aire at a selected number of sites and sampling of patients in clinics considered. Patients and carers would be only too happy to cover this.<br><br>I wonder if the research team would consider presenting their data at national expert conferences, they may be able to convene a parallel meeting or similar activity to aid their interaction with clinicians and AHP's<br><br>ii. I see no issue<br><br>iii. The main strength is the quality of the research team (DR and KG for the SR and MA; EW for working with patients/carers; PCUK team for their support and to raise the impact of the work) combined with the topic which is an area of unmet need; main downside is the need to ensure that the data and conclusions are generalisable<br><br>iv. A SR and MA is not very novel but there is clear need here

Has the research been designed with reference to an appropriate review of the existing literature?
Yes

**Work plan and proposed management arrangements**<br>i) How appropriate are the work plan and project management arrangements, and do they give confidence that proposed milestones will be met within the specified timeframe?<br>ii) Are the necessary clinical, academic or organisational links needed to support the research, in place?<br>iii) Please assess whether the key risks identified by the applicants have been adequately addressed, such as:<br><br>• Ethical, scientific, technical and organisational challenges<br>• Intellectual Property (IP) and commercial issues<br>iv) Please identify any additional barriers to the proposed work, not mentioned in the application form, that the applicants are likely to encounter in meeting their milestones<br>v) If this application has been submitted to the NIHR Programme Grants for Applied Research Programme, what added value would be delivered over and above the dividends from the individual elements?
i. no issues for me<br><br>ii. yes<br><br>iii. yes<br><br>iv. none I can think of<br><br>v. na

**Plain English summary**<br>The plain English summary is intended for an interested audience, who are not necessarily specialists. The summary should be written at roughly the same level as an article in a newspaper. With this in mind, please

comment on the following:<br><br>i) Does the plain English summary give a clear explanation of the research?<br>• Does it help you carry out your review? If not, why not?<br>• Is the language used appropriate and clear? If not, where are the problems?<br>• Are scientific terms, abbreviations and jargon explained? If not, which terms need explanation?<br>ii) If this research is funded, the plain English summary will be published on a variety of websites, without the rest of this application form. Could this plain English summary be used on its own to describe the proposed research? If not, what further information is needed?<br><br>Further information for researchers on how to write a plain English summary and what to include in a summary is available online at NIHR Make it clear http://www.invo.org.uk/makeitclear/.
i. the PES is well written<br><br>ii. it could

4. Strength of the research team

i) How well are the roles of the team members described? Is the overall team well coordinated?<br>ii) On the basis of track record in relevant areas, how qualified are the applicants to undertake the work using the methodologies proposed?<br>iii) To what extent does the research team have the necessary breadth and depth of expertise to deliver the proposed work (*e.g.* as judged by publication output and previous research funding)?<br>iv) How could the strength of the research team be improved?<br>v) If the lead applicant is inexperienced, does he/she have appropriate support (*e.g.* from their organisation and/or more senior colleagues) to deliver the work plan?
I. I covered this above; this is a very strong team<br><br>ii. highly<br><br>iii. no issues<br><br>iv. I am not sure that Prof Eila Watson has enough time allocated to the work<br><br>v. not an issue

5. Impact of the proposed work

**Dissemination, outputs and anticipated impact**<br>NIHR aims to fund research that has the potential to be of significant benefit to the NHS, patients and the public. To support this, the applicants should consider how they will achieve impact from the outset. This helps them to identify potential impact and beneficiaries, and plan processes by which the research can directly, or incrementally over time, lead to change.<br><br>i) Have the applicants clearly expressed the problem and outlined how this research contributes towards a solution? Are the planned outputs appropriate and sufficient?<br>ii) Is it clear from the application who or which groups (including, as appropriate, healthcare planners, clinicians, patients and/or policy makers) are expected to benefit the most, how they will be engaged and communicated with, and if appropriate methods of engagement and dissemination are planned?<br>iii) Have the applicants set out appropriate activities and resources to achieve their impact goals? Is there a

realistic trajectory and estimate of timescales for the benefit to reach patients/ public/ health and care services? Are there clear connections between outputs, engagement processes and impact goals?<br>iv) Have the applicants clearly considered what follow-on support they might need to generate or upscale impact, and how they might leverage further investment?<br>v) Have the applicants sufficiently and correctly identified any potential barriers they might face (e.g. IP, regulatory and acceptability) and have they properly considered how these may be overcome?<br>vi) Have the applicants clearly and realistically outlined the anticipated impacts, the likely scale of these impacts (both in the shorter and longer terms), and the sequence in which the impacts might occur?
i. yes<br><br>ii. it is<br><br>iii. yes, though I think engaging with expert national groups/societies would give an opportunity to both share data with from WP1 as part of WP2 and to raise awareness of this work<br><br>iv. the involvement of PCUK is key here<br><br>v. yes<br><br>vi. I dont think this is an issue. This data sythesis is needed and outputs will have a positive impact upon recommendations for treatment and future research

6. Value for money

i) Taking into consideration the costs associated with undertaking the research, is there sufficient justification for the resources requested? Are all costs essential for the work proposed?<br>ii) Are appropriate resources set aside for patient and public involvement - including plans for a training and support budget?<br>iii) Where relevant, are resources to support impact - other than those for public and patient involvement - included and appropriate?<br>iv) Taking into account the total cost of the research, including the NHS costs, to what extent does the research provide value for money?<br>v) If required, are funds requested for NHS support and treatment costs appropriate and justified?
i. I find that this small number of SR and MA to be poor value - though I am sure that this is the 'going rate'. In comparison, the contributions by EW and the PCUK team are what will make this study a success, and their cost is minor<br><br>ii. yes<br><br>iii. yes<br><br>iv. Given that much is spent on novel therapies for PC with slow progress, this project represents value as it will lead to direct impact and will shape future work<br><br>v. na

7. Involvement of patients and the public

**Was there any patient and public involvement in the application?**	Yes

i) What is your assessment of the patient and public involvement (if any) in the development of the application, including involvement in: Identifying the research topic; prioritising the research questions; preparing the application (e.g. contributing to the research design); and identifying potential impact?<br>ii) What is your assessment of any proposed plans for patient and public involvement throughout the life of the research? Can you identify particular strengths, weaknesses and/or areas for improvement?
i. It has been appropriate. Involvement of PCUK ensures that the issue is highly relevant<br><br>ii. It is appropriate though see my concern re the sampling method for patients in WP2

8. Additional Comments

If you have any additional comments, not covered by the sections above, please provide them below.

NIHR | National Institute for Health Research

Research for Patient Benefit Review Summary

Reference Number	NIHR202727
Research Title	Management of pain and cachexia in pancreatic cancer: Systematic reviews of randomised controlled trials and network meta-analysis
Chief Investigator	Dr Danielle Roberts
Contracting Organisation	University College London Hospitals NHS Foundation Trust
Total Amount Requested	£148,724.00
Reviewer Number	2

Table Of Contents

1. Reviewer Expertise
2. Relevance of the proposed research
3. Quality of the proposed work
4. Strength of the research team
5. Impact of the proposed work
6. Value for money
7. Involvement of patients and the public
8. Additional Comments

1. Reviewer Expertise

**Please indicate the nature of your expertise by clicking on the appropriate tick box(es) below:**
Clinician in a broadly related field, Researcher in a broadly related field, Other (please see below)

If the tick boxes above do not adequately capture the nature of your expertise, please briefly provide details in the box below (or use it to give us more detail about your expertise if you wish):
A Consultant Surgeon who treats patients with pancreatic cancer, chairs pancreatic cancer MDT and undertaking research in the field of pancreatic cancer.

2. Relevance of the proposed research

i) How relevant and important is the proposed research to the priorities and needs of the NHS, and does it offer a health/healthcare solution with demonstrable benefit to patients?<br>ii) Does the application demonstrate an awareness and understanding of previous relevant research or developments in this area?<br>iii) To what extent does the proposed research add distinct value or advance existing knowledge in this area, taking into account wider ongoing or completed research?
1. Pancreatic cancer management is an unmet need. The research is relevant to patients with pancreatic cancer as both pain and cancer cachexia are common problems experienced by patients with pancreatic cancer. Clear recommendations regards ideal management pathways could significantly improve the quality of life for the patients with pancreatic cancers.<br><br>2. The application provides a comprehensive review of literature. However the proposal does not take into consideration utilisation of radiotherapy for management of pain in patients with pancreatic cancer.<br><br>3. The proposal would synthesise the current evidence and add to the literature

3. Quality of the proposed work

Research Design
i) How appropriate is the research design in relation to the stated objectives?
ii) To what extent is the proposed design and methodology for all elements of the research well defined, appropriate, valid and feasible within the timeframe and resources requested?
iii) What are the strengths and weaknesses of the research design as proposed?
iv) To what extent does the research show originality and innovation?

A Network meta-analysis would help compare the different treatment options for patients. The innovative combination of appraising the literature and using focus groups will provide high quality care regards the interventions and the acceptance to patients and carers. Patients with pancreatic cancer have a poor prognosis with poor functional status. Using focus groups of patients and carers - acceptability and feasibility of the proposed interventions would be valuable. However the authors have not justified the sample size for the focus groups.

The weakness of the proposal is a failure to consider other modalities of pain relief such as radiotherapy and focused ultrasound therapy. There is also no discussion regards scientific sample size calculation for surverys and focus groups part of the study.

Has the research been designed with reference to an appropriate review of the existing literature?

Yes

Work plan and proposed management arrangements
i) How appropriate are the work plan and project management arrangements, and do they give confidence that proposed milestones will be met within the specified timeframe?
ii) Are the necessary clinical, academic or organisational links needed to support the research, in place?
iii) Please assess whether the key risks identified by the applicants have been adequately addressed, such as:

- Ethical, scientific, technical and organisational challenges
- Intellectual Property (IP) and commercial issues

iv) Please identify any additional barriers to the proposed work, not mentioned in the application form, that the applicants are likely to encounter in meeting their milestones
v) If this application has been submitted to the NIHR Programme Grants for Applied Research Programme, what added value would be delivered over and above the dividends from the individual elements?

The proposed work plan is achievable in the time scale proposed. There is suitable organisational support.

Plain English summary
The plain English summary is intended for an interested audience, who are not necessarily specialists. The summary should be written at roughly the same level as an article in a newspaper. With this in mind, please comment on the following:

i) Does the plain English summary give a clear explanation of the research?

- Does it help you carry out your review? If not, why not?
- Is the language used appropriate and clear? If not, where are the problems?
- Are scientific terms, abbreviations and jargon explained? If not, which terms need explanation?

ii) If this research is funded, the plain English summary will be published on a variety of websites, without the rest of this application form. Could this plain English summary be used on its own to describe the proposed research? If not, what further information is needed?

Further information for researchers on how to write a plain English summary and what to include in a summary is available online at NIHR Make it clear http://www.invo.org.uk/makeitclear/.

The plain summary in English clear to understand

4. Strength of the research team

i) How well are the roles of the team members described? Is the overall team well coordinated?<br>ii) On the basis of track record in relevant areas, how qualified are the applicants to undertake the work using the methodologies proposed?<br>iii) To what extent does the research team have the necessary breadth and depth of expertise to deliver the proposed work (*e.g.* as judged by publication output and previous research funding)?<br>iv) How could the strength of the research team be improved?<br>v) If the lead applicant is inexperienced, does he/she have appropriate support (*e.g.* from their organisation and/or more senior colleagues) to deliver the work plan?
Having a Co-PI would provide significant support to the PI. The PI has an excellent track record of synthesis of evidence. The team is balanced and well placed to deliver on the project. Support from a major pancreatic cancer charity would help deliver the project

5. Impact of the proposed work

**Dissemination, outputs and anticipated impact**<br>NIHR aims to fund research that has the potential to be of significant benefit to the NHS, patients and the public. To support this, the applicants should consider how they will achieve impact from the outset. This helps them to identify potential impact and beneficiaries, and plan processes by which the research can directly, or incrementally over time, lead to change.<br><br>i) Have the applicants clearly expressed the problem and outlined how this research contributes towards a solution? Are the planned outputs appropriate and sufficient?<br>ii) Is it clear from the application who or which groups (including, as appropriate, healthcare planners, clinicians, patients and/or policy makers) are expected to benefit the most, how they will be engaged and communicated with, and if appropriate methods of engagement and dissemination are planned?<br>iii) Have the applicants set out appropriate activities and resources to achieve their impact goals? Is there a realistic trajectory and estimate of timescales for the benefit to reach patients/ public/ health and care services? Are there clear connections between outputs, engagement processes and impact goals?<br>iv) Have the applicants clearly considered what follow-on support they might need to generate or upscale impact, and how they might leverage further investment?<br>v) Have the applicants sufficiently and correctly identified any potential barriers they might face (e.g. IP, regulatory and acceptability) and have they properly considered how these may be overcome?<br>vi) Have the applicants clearly and realistically outlined the anticipated impacts, the likely scale of these impacts (both in the shorter and longer terms), and the sequence in which the impacts might occur?
There is a clear plan for dissemination for patients & their clinicians. This is likely to have impact on management pathways and provide evidence for NICE guidelines. Patients and their carers are likely to benefit the most and there could be cost savings to NHS by using the best available evidence.

6. Value for money

i) Taking into consideration the costs associated with undertaking the research, is there sufficient justification for the resources requested? Are all costs essential for the work proposed?<br>ii) Are appropriate resources set aside for patient and public involvement - including plans for a training and support budget?<br>iii) Where relevant, are resources to support impact - other than those for public and patient involvement - included and appropriate?

iv) Taking into account the total cost of the research, including the NHS costs, to what extent does the research provide value for money?<br>v) If required, are funds requested for NHS support and treatment costs appropriate and justified?
The project would be good value for money. It would rank the available modalities of treatment for pancreatic cancer pain and cachexia for a cancer with poor prognosis and unmet need. It would also identify areas for future research.

7. Involvement of patients and the public

**Was there any patient and public involvement in the application?**	Yes

i) What is your assessment of the patient and public involvement (if any) in the development of the application, including involvement in: Identifying the research topic; prioritising the research questions; preparing the application (e.g. contributing to the research design); and identifying potential impact?<br>ii) What is your assessment of any proposed plans for patient and public involvement throughout the life of the research? Can you identify particular strengths, weaknesses and/or areas for improvement?
A patient and family member with pancreatic cancer and a pancreatic cancer charity have provided input and PPI work has been undertaken through PCUK

8. Additional Comments

If you have any additional comments, not covered by the sections above, please provide them below.
Pancreatic cancer management is an unmet need with most patients not having curative options. Symptom control such as pain and cachexia are the mainstay of treatment in majority of the patients. The proposal synthesises the available evidence. This proposal is from a research group with a very strong track record of synthesising evidence by undertaking Cochrane reviews. They are well placed to undertake network meta-analysis. Excluding radiotherapy in evaluation would be a significant weakness in the technique used to evaluate the evidence as radiotherapy is a commonly used technique.

NIHR | National Institute for Health Research

Research for Patient Benefit Review Summary

Reference Number	NIHR202727
Research Title	Management of pain and cachexia in pancreatic cancer: Systematic reviews of randomised controlled trials and network meta-analysis
Chief Investigator	Dr Danielle Roberts
Contracting Organisation	University College London Hospitals NHS Foundation Trust
Total Amount Requested	£148,724.00
Reviewer Number	3

Table Of Contents

1. Reviewer Expertise
2. Relevance of the proposed research
3. Quality of the proposed work
4. Strength of the research team
5. Impact of the proposed work
6. Value for money
7. Involvement of patients and the public
8. Additional Comments

1. Reviewer Expertise

**Please indicate the nature of your expertise by clicking on the appropriate tickbox(es) below:**
Member of public with a more general view, Patient or service user with direct experience of this area

If the tick boxes above do not adequately capture the nature of your expertise, please briefly provide details in the box below (or use it to give us more detail about your expertise if you wish):
Lay member whose grandad died from pancreatic cancer

2. Relevance of the proposed research

i) Is there a clear and credible reason for doing this research? If there is, what is it?<br>ii) Is this research important or relevant to patients or carers? Why is that?<br>iii) Could the results of the research make a difference to patients or carers? If yes, how would they make a difference? If not, why not?
The rise of pancreatic cancer in the UK and indications of it being the third most common cause of death and associated symptoms including pain, cachexia, and jaundice and the resulting uncertainty around how best to address these, form part of the rationale for this study. The primary endpoints of the study concern a systematic review, so it would be good to see how the secondary endpoints which do include qualitative methodology pick up patient views. The statement that this will *"help stream and lead to more research in areas where there is an evidence gap"*, is articulated throughout the proposal but additional clarity on how this will occur as a result of this piece of work may strengthen the proposal more. As a person whose grandfather died from pancreatic cancer, I would like to see more explanation of what the existing pathway is, what evidence means and why it would have mattered to my family and what the other issues are around context of treatment and how this fits into emerging clinical research trials abroad articulated from the very start. The outcomes are relevant to patients and their carers though the actual explanation given could be stronger. The detail given in pages 9-14 explains the nature of the symptoms associated with pancreatic cancer and cachexia and pain though my own personal lived experience of my grandfather's journey would suggest that there are also other outcomes that represent variables that should be included in this proposal such a sleep and emotional wellbeing, effects on finance. The primary endpoint is to look at safety and efficacy of different treatments through network meta-analyses, why not include PROMS and PREMs in this too? The study will initially conduct a systematic review and NMA looking at patients with unresectable pancreatic cancer and those without and examining a range of interventions which is broad in nature: NSAIDs, opioid analgesics, neuropathic pain medications, thoracoscopic splanchnectomy, celiac plexus blocks or

neurolysis, splanchnic nerve blocks or neurolysis, pancreatic duct stenting, acupuncture, and hypnosis. The second review will look at pancreatic enzyme replacements, increased calorie intake, ketogenic diet, amino acid supplementation, nutritional supplements (oral or parenteral), appetite stimulants, corticosteroids, NSAIDs, progesterone analogues, cytokine inhibitors, omega-3 fatty acids, and exercises to improve muscle mass. Later in the proposal the differences between patient reported quality of life metrics and medication is mentioned which is good to see, given that any given medication may have better survival or efficacy outcome but cause more side effects, I would like to seeare a stage 3-4 patient you may prefer a medication that has an immediate effect on your quality of life metric e.g energy management or nausea that given day than something that has the potential to prolong your life but still carries with it significant side effects, so I would have preferred to have seen this emphasis on QoL PROMS, PREMs as part of the primary end point part of the study.

3. Quality of the proposed work

Research Design
i) Are the outcomes the researchers are planning to measure appropriate? Will the research ultimately benefit patients, service users, carers and/or the public? Why is that the case? Are there other outcomes that are more important? If so, what are they?
ii) Have the researchers taken a realistic approach to recruiting people to participate in their research? Could this be improved and if so, how? Do you think people are likely to agree to take part? Would you be willing to take part in the research or suggest to a friend that they did?

The PI has taken into account previous comments concerning the running of focus groups and detailed description of this is contained. I think people would be willing to take part in these and support from the charity mentioned and NCRI is a plus, though the limited numbers (50) taking part in the actual focus groups is a shame. Overall understanding stakeholder experience is an explicit goal of the project, which is again commendable and reflects an' understanding of the importance of human factors and PPI and the applicants have clearly worked hard to engage with those that have a developed a network through which they can easily engage patients as necessary. The inclusion of a mixed focus group (50% patients/carers and 50% healthcare professionals) will enhance transparency and is a strength, as is ensuring that the feasibility of treatments in NHS for each of pain and cachexia (after stratification by the category of healthcare professionals) will be presented in two ways, ordered by the number of people who ranked an intervention as the most feasible treatment and then by their weighted ranking score using weights applied in reverse. I do wonder if some sort of Delphi methodology would be effective or if the inclusion of additional insight from tools that use social media inferred to would be an added bonus, an example of this is the VOICE platform. The differentiation between how different solutions can be applied within primary and community care settings is notable and topical given current transformation agenda for the NHS with the integrated care systems announced recently. Why does the study refer to patients in Germany? Their healthcare system is structured differently and funded differently than in the UK, is this a comparative reasonable model to take for someone having the UK NHS experience in terms of time frames and different stages of the diagnostic pathway? Is the effect of the pandemic taken into account, as to how this may affect recruitment or treatment pathways? As a former individual family member of a person with pancreatic cancer, I would not hesitate to recommend to others to take part in supporting this study and any work in pancreatic cancer research in general but would have liked to have seen more facilitation at earlier stage and patient views contained as an actual primary end point objective not secondary.

Has the research team taken account of previous research in this area?	Yes

Work plan and proposed management arrangements
i) How are any plans for patient and public involvement in the research also referred to in the work plan and in the proposed management arrangements? Could the plan and the management arrangements be improved from this perspective?

The overall work plan is mapped out throughout the project and it is evident that it fits in well against the overall framework of the planned milestones, but though there is suggestion of PPI, this is not actually contained in the actual GANT chart submission included in the Annexes. Work package 1 sees a systematic review and NMA looking at patients with unresectable pancreatic cancer and those without and examining a range of interventions

which is broad in nature. It would be nice to have a more detailed explanation of what counts as a "*secondary analysis*"? The creation of separate patient advisory group and patient steering group committee is noted and ensures appropriate governance but am concerned that some patients with less experience or familiarity with how research works, may not feel comfortable expressing their views in front of clinical staff in the actual focus group and may benefit from additional training. Overall, the proposed management structures are sound but would be nice to get more clarity on what the difference is between the activities that the research management and research steering committee do? The proposal work plan includes division of tasks and distribution in people hours to deliver aspects of the work which is detailed and sub-divided into tasks seen in the GANT chart. Additional allocation of time on risk mitigation could be useful given the current situation in the NHS with the backlog of work as a result of the pandemic, one wonders if the second co-lead applicant who has the most familiarity with such large grants and is also a practising NHS consultant, will have enough time to effectively support the primary applicant and if this is realistically reflected in the current task and time division, more assurance would make the application even more convincing. The second WP involves examining the development of a clinical care pathway, and it is excellent that they are including the carer perspective, though I do wonder if it would have been possible to ask patients what questions they wanted to see in the initial survey or use some other prior work instead of relying solely on one individual to develop the questions, even though this individual has substantial prior experience in conducting surveys and even though these will be reviewed by the co-applicants including the PPI and qualitative researcher. There are companies such as the NHS Innovation Accelerator backed Patient Experience Platform that use AI and predictive analytics to pick up social insight why not use something like that to get a broader and more representative viewpoint across in the study in the way that would reflect views on the pathway from a bigger number of people and/or this could also be integrated into the existing social media channels used by some of the charitable partners.

Plain English summary
The plain English summary is intended for an interested audience, who are not necessarily specialists. The summary should be written at roughly the same level as an article in a newspaper. With this in mind, please comment on the following:

i) Does the plain English summary give a clear explanation of the research?

- Does it help you carry out your review? If not, why not?
- Is the language used appropriate and clear? If not, where are the problems?
- Are scientific terms, abbreviations and jargon explained? If not, which terms need explanation?

ii) If this research is funded, the plain English summary will be published on a variety of websites, without the rest of this application form. Could this plain English summary be used on its own to describe the proposed research? If not, what further information is needed?

Further information for researchers on how to write a plain English summary and what to include in a summary is available online at NIHR Make it clear http://www.invo.org.uk/makeitclear/.

The plain English summary is good but there is still some technical language used that needs to be modified such as *"evidence-based clinical care pathways", "RCTs", "advanced methods".*How is *"destruction of the nerves behind the pancreas"* a treatment what does this mean and how do they do it? Can you insert a sentence that explains why there is uncertainty as to which treatments work for cachexia and why this is a problematic area? Has there not been research on this before? Do they have very unpleasant side effects that create an additional burden on the patient? Do all pancreatic cancer patients get it? If not, why not? Can you include a glossary of terms that lists technical language like RCTs? Consider removing the "*when conducted well"* as that is a subjective assessment open to interpretation, it also create an element of doubt in the average lay that may assume that they always do work well, you also fail to explain the need to assesses these RCTs and/or should provide an explanation as to what a "*standard tool"* is, and why it matters to the way clinical research is conducted from the patients' view point. Does a patient need to know what an "*advanced method"* is? So what? The sentence that talks about the charity being able to influence policy makers and *"effectively advocate for optimal treatment approaches to be adopted by the NHS"* whilst admirable needs to be validated by a demonstrable and measurable example and/or may also be misleading without reference to HTA and other assessments that usually support any efficacy/pathway optimisation analysis, I would consider amending or removing this altogether if the purpose of including it is to convey to the lay public that one of the outputs or consequences of this study is to change the way that patients receive treatment, simplify and explain the rationale and what your primary short and long term objectives are that could integrate into such a campaign.

4. Strength of the research team

i) Does the research team appear to have the right mix of skills to carry out this research? For example, if the research involves looking at what nurses do, is there a nurse on the team? If not, how could the team be strengthened?<br>ii) Is there one or more suitably experienced member of the research team with responsibility for coordinating, supporting and delivering patient and public involvement activities? If not, how could this be addressed?<br>iii) Are patients, service users or carers included in the research team? And if so, is it clear what their role or roles will be and what they will bring to the research team?
The lead applicant is new to undertaking large grants which is a cause of concern that is somewhat mitigated by the inclusion of an experienced co-lead and his track record on similar type NIHR grants for Cochrane systematic reviews that also include liver and pancreatic diseases. The lead PI does not have experience in undertaking financial management or co-ordination of reviews at this level, but the willingness to learn is admirable. The addition of a qualitative methods researcher who has experience in conducting surveys and running focus groups is a welcome improvement as is the support of the research nurse part-time. The overall mixture of skills in the consortium is fairly well balanced with experience in Cochrane systematic reviews, network meta-analysis, project management in NIHR grants that have led to policy and implementation science change, qualitative methodology and experience of patient support as a charitable entity. The team could be strengthened by including the usage of technology enabled tools and platforms such as VOICE platform or Patient Experience Platform, the latter an NHS Innovation Accelerator start-up that uses AI and predictive analytics to pick up a broader representative range of patient insight and views that can add further value to the channels used by the charity partner. One does wonder why not also reach out to established patient groups outside of the Uk, such as European Cancer Patient Coalition or Pancreatic Cancer Europe that have a very broad network. The inclusion of both the Head of Research at Pancreatic Cancer UK (PCUK) and a research manager from the same charity are strengths as the research manager, Anna Lakey, is likely to be instrumental to the delivery of the project. There isn't a lot of detail provided on this individual's past background in terms of CV or past projects. Patient and public involvement activities will be supported by both the lay dedicated member as well as the creation of a PAG . The focus group discussions will be led by an experienced qualitative researcher, Prof Ella Watson and facilitated by PCUK itself. The co-PI, who we assume must mean Prof Watson, is stated as having used *"the approach of focus group discussions informed by survey results(of patients and healthcare professionals) in the design of research".* The applicants expressly very clearly how the focus groups will take place, but less so how and what the overarching strategy for PPIE is in terms of short, medium and long-term objectives. What kind of training will they provide lay or patient members? How will they work with the UCL Centre for Co-production and National Voices to address health inequality in terms of engagement with BAME communities? How will they deal with conflict management if it arises? The inclusion of a lay PPI member, Mrs Sarah Prideaux, that has experience as a carer of someone living with pancreatic cancer will contribute patient and lay perspective into what patients think of the project's research direction and patient outcomes.

5. Impact of the proposed work

**Dissemination, outputs and anticipated impact**<br>NIHR aims to fund research that has the potential to be of significant benefit to the NHS, patients and the public. To support this, the applicants should consider how they will achieve impact from the outset. This helps them to identify potential benefits and beneficiaries beyond the academic community, and plan processes by which the research can directly or incrementally over time, lead to change in the 'real world'.<br><br>i) Have the applicants clearly expressed a real-world problem and how their research contributes towards a 'solution'? How well do the planned outputs match this aim? If not, what changes are needed?<br>ii) Have the applicants made it clear what impacts they are aiming to achieve from the research? Are these plans appropriate? Are they achievable? Do they seem realistic in terms of scale and timing? If not, what needs changing?<br>iii) Have the applicants clearly stated who will benefit from this research (e.g., patients, carers, clinicians, policy makers, healthcare planners) and how they will benefit? Are plans to engage and communicate with these individuals/groups appropriate? If not, what is missing?

iv) Have the applicants chosen suitable activities to achieve impact? Have they made it clear how the outputs, beneficiaries and planned impacts are linked? If not, what needs changing?<br>v) Are the applicants clear on what would be needed (e.g. more funding, further partnerships) to sustain or increase impact after the project? If not, what else needs to be considered?<br>vi) Have the applicants sufficiently considered the barriers they may face in achieving impact (e.g. regulations, intellectual property/rights, acceptability to users)? Have they adequately considered how to overcome these? If not, what is missing?
The inclusion of the lay member is a good way to cross-refer and ensure validity of the outcomes chosen in the work, which are based on the Core Set of Patient-reported Outcomes in Pancreatic Cancer (COPRAC) study. As an individual whose grandfather died from pancreatic cancer I would have thought other outcomes could also play a role here such as sleep quality and mobility. My grandfather was an insomniac both as a result of unmanaged pain due to being a fast metabolizer of some medications/ side effects and more mentioning of outcomes that are financial in nature, such as loss of income and/or disruption to employment and the effect on mental health on the carer and patient's family could also be relevant. The applicants intend to use systematic reviews and NMA to get better quality information on the management of pain in people with pancreatic cancer and through this can better examine if there are any interactions between different combinations of treatments. They refer to insight provided by the PCUK clinical network and the NCRI Upper Gastrointestinal Group (Pancreatic cancer Workstream) so the necessary stakeholders are engaged (see letters of support). They have well established links with NHS England Cancer Policy Team, NHS Cancer Programme Team, Cancer Alliance, suggests broader ambitions to engage with different policy forums including the NHS Hepatobiliary and Pancreas Clinical Reference groups. What about prioritising the usage of online tools to better embed and bring in different patient views?I would like to see detailed marketing communications dissemination plan by the charitable entities as to how they intend to communicate their findings to different audiences as regulatory authorities, media and commissioning groups may need a differentiated messaging strategy. How will carers benefit from this work and how can a HTA or other health economics based analysis that can compare some of the therapeutic interventions in terms of QYALY, DALY etc be used? The risk mitigation plan focuses on the rationale of the research objectives there is no mention of IP, licensing or rights per se. Freedom to operate analysis has not been undertaken and there is no indication of any commercial, financial projections or similar considerations but then the scope of the proposal may not apply in this area. One assumes that the actual scientific findings will be shared through various prestigious medical bodies and journals but there is no mention of which.

6. Value for money

The NIHR provides guidance on what can and cannot be included in the costs of research. CCF carries out an initial financial scrutiny of all applications received. A more detailed scrutiny of finances is always carried out on any application that is recommended for funding.<br>As a public reviewer, you are not expected to assess whether the entire research budget is costed correctly. However, comments on the following aspects are welcome:<br>i) Overall, does the research budget seem a reasonable investment of public money? Could it save health and social care costs in the long term?<br>ii) Are the resources set aside for patient and public involvement appropriate for the proposed activities? E.g. for training and support, travel and other expenses, staff salaries? For more see: INVOLVE's Involvement Cost Calculator. If not, how could it be improved?
Overall, the allocation and justification of resources are appropriate and the costs are described in detail and justified, allocated well and linked to the goals of the project. The budget allocation of approximately £150k seems fair and reasonable and is compared to the costs of running a randomised controlled trial. I do wonder if there are other existing tools and surveys linked to broader research on pain that could have been combined to better address "*the uncertainty in the management of pain in pancreatic cancer"* and so wonder if they can include more information on the actual *"costs of providing supportive care for people with pancreatic cancer"* and how this project can support incremental cost savings. Given how the recent pandemic has demonstrated fully online delivery of training conferences, is the PPI facilitation fee for £3152.00 travel justified? Could the £2400 allocated for focus groups be reduced through usage of some sort of online and digital tools? PPI costings are in accordance with INVOLVE criteria, with 4 meetings costed at £576 and patient representatives at 0.5 hours/week x 2 patient representatives x 104 weeks x £25 per hour, bringing it to £2496.00 so the overall budget is well within what I would expect for a project of this size, and thus reasonable value for money. In addition to NIHR INVOLVE why not refer to the UNICEF minimum quality standards and indicators in community engagement too?

7. Involvement of patients and the public

**Was there any patient and public involvement in the application?**	Yes

i) What is your assessment of the patient and public involvement in the development of the application including involvement in: identifying the research topic; prioritising the research questions; preparing the application (*e.g.* contributing to the research design); and identifying potential impact?<br>ii) What is your assessment of any proposed plans for patient and public involvement throughout the life of the research? Can you identify particular strengths, weaknesses and/or areas for improvement?
A notable strength as it appears that they hosted a workshop which also included patients' views incorporated into the research topic, though additional detail on additional measures that could be taken to prioritize certain pints would strengthen the application further. For the development of the full application the project has not only tapped into the existing relationships they developed with patients and charities in the space that have great patient support and outreach, but has also sought input through the creation of focus groups. These focus groups are a mix of patients, their carers, and healthcare professionals and form a basis that will develop the care pathway and will cover two focus group discussions (involving 8 to 10 participants each) and each symptom. This will enable a balance of views and patients will be directly asked to contribute to asses the acceptability of each intervention in the order of preference for each of pain and cachexia and the actual survey questions will be reviewed by the PPI member but in my opinion this is a slight weakness and I would prefer to see the integration of many patient views into the actual design of these survey questions themselves from the start, as opposed to just the review stage. A strength of the application is that the researchers will seek input into the lay summary including from the lay member, to ensure the content is easy to comprehend, but I would like to see more mention on how they will go about addressing accessibility needs and increasing diversity in the views presented across. I do wonder if the use of the VOICE platform https://www.voice-global.org/ which some patients like could be of value and what about something like https://miro.com/ to create joint sessions in working groups and/or infographics and video explainers to be able to better explain what a systematic group and/or a café scientifique is to the broader public? I also wonder if using a combination of modalities and resources for training would enable better rapport between the researchers and patients via online exercises e.g pub quiz (social), lego building games (facilitated) and/or using short snippets on TikTo to engage younger audiences could be of use. Patients are included throughout the lifecyle of the project but this could be further strengthened by having reporting evaluation tools in place that measure the effectiveness of the PPIE activities in addition to the GRIPP2 framework which have been developed by industry EU funded projects such as PFMD.<br><br>It would be great to see how new digital technologies can be better embraced to co-design future materials and how participatory methods can further be used to facilitate priority setting, co-design of new content and refinement of work done.

8. Additional Comments

If you have any additional comments, not covered by the sections above, please provide them below.
The activities chosen by the applicants and the timescale for the proposed work seems fine. The applicants are clear that patients want more information on pain but there is less information as to how their carers and the NHS will benefit. The explanation could be clearer as could the explanation of what they will do to sustain and increase the impact after the project, they simply refer *"providing information on the relative effectiveness of different treatments in the reviews and a clinical care pathway for the management of pain and for prevention and treatment*

of cachexia" as well as *"research recommendations based on the systematic reviews".* It would be good if they could explain what this means in the overall context of commissioning and integrated care systems. Whilst there is no doubt that the charity and the NCRI Pancreatic Workstream are great partners to have involved in the project, there is insufficient detail of a medium-term plan that would show how the impact on this work can create differentiated service delivery and I would like to see how it can be used to create impetus for more research with industry.

Why is there no Work package dedicated to PPI within the GANT chart? Would have liked to have seen more PROMs and PREMs accounted for in the final report and/or survey involvement by charities and patients and their carers!

1 page of references only minimal!

Pain For example, pain may be reported using the visual analogue scale in one trial, Brief Pain InventoryShort Form, and verbal rating scale in another trial why not use something like an online survey tool?

Why is this based on the JLA-PSP on pancreatic cancer' in Germany?

NIHR | National Institute for Health Research

Research for Patient Benefit Review Summary

Reference Number	NIHR202727
Research Title	Management of pain and cachexia in pancreatic cancer: Systematic reviews of randomised controlled trials and network meta-analysis
Chief Investigator	Dr Danielle Roberts
Contracting Organisation	University College London Hospitals NHS Foundation Trust
Total Amount Requested	£148,724.00
Reviewer Number	4

Table Of Contents

1. Reviewer Expertise
2. Relevance of the proposed research
3. Quality of the proposed work
4. Strength of the research team
5. Impact of the proposed work
6. Value for money
7. Involvement of patients and the public
8. Additional Comments

1. Reviewer Expertise

**Please indicate the nature of your expertise by clicking on the appropriate tick box(es) below:**
Clinician in a broadly related field

If the tick boxes above do not adequately capture the nature of your expertise, please briefly provide details in the box below (or use it to give us more detail about your expertise if you wish):

2. Relevance of the proposed research

i) How relevant and important is the proposed research to the priorities and needs of the NHS, and does it offer a health/healthcare solution with demonstrable benefit to patients?<br>ii) Does the application demonstrate an awareness and understanding of previous relevant research or developments in this area?<br>iii) To what extent does the proposed research add distinct value or advance existing knowledge in this area, taking into account wider ongoing or completed research?
1) Undoubtably high priority, disease of increasing incidence with poor symptom control and lack of good guidance.<br><br>2) Yes, it is specifically looking to study previous high impact studies, i.e RCTs<br><br>3) There are currently no NMAs in this field. It is important that we have comparative effectiveness research to allow us to create guidelines that understand the relative effectiveness of different treatments

3. Quality of the proposed work

**Research Design**

i) How appropriate is the research design in relation to the stated objectives?<br>ii) To what extent is the proposed design and methodology for all elements of the research well defined, appropriate, valid and feasible within the timeframe and resources requested?<br>iii) What are the strengths and weaknesses of the research design as proposed?<br>iv) To what extent does the research show originality and innovation?
1) NMA is appropriate to summative evidence.<br><br>2) I have some concerns about the heterogeneity of the outcomes the team will encounter, survival is relatively straight forward but by the teams own admission PROMs is difficult and definitions of cachexia variable.<br><br>3) NMA is is the best approach for evidence synthesis and the team has a strong background. There maybe issues with transitivity in the studies, in some ways this will only be known when it is done<br><br>4) As a first of its kind it is original in the field although well estd more broadly.

Has the research been designed with reference to an appropriate review of the existing literature?
Yes

**Work plan and proposed management arrangements**<br>i) How appropriate are the work plan and project management arrangements, and do they give confidence that proposed milestones will be met within the specified timeframe?<br>ii) Are the necessary clinical, academic or organisational links needed to support the research, in place?<br>iii) Please assess whether the key risks identified by the applicants have been adequately addressed, such as:<br><br>• Ethical, scientific, technical and organisational challenges<br>• Intellectual Property (IP) and commercial issues<br>iv) Please identify any additional barriers to the proposed work, not mentioned in the application form, that the applicants are likely to encounter in meeting their milestones<br>v) If this application has been submitted to the NIHR Programme Grants for Applied Research Programme, what added value would be delivered over and above the dividends from the individual elements?
1 The team is led by clinicians experienced inconducting systematic reviews and network meta-analysis. The milestones appear reasonable.<br><br>2. Yes the inclusion of Prof Kurinchi Gurusamy adds to the skills/experience of the team<br><br>3/4/5. No issues identified

**Plain English summary**<br>The plain English summary is intended for an interested audience, who are not necessarily specialists. The summary should be written at roughly the same level as an article in a newspaper. With this in mind, please comment on the following:<br><br>i) Does the plain English summary give a clear explanation of the research?<br>• Does it help you carry out your review? If not, why not?<br>• Is the language used appropriate and clear? If not, where are the problems?<br>• Are scientific terms, abbreviations and jargon explained? If not, which terms need explanation?<br>ii) If this research is funded, the plain English summary will be published on a variety of websites, without the rest of this application form. Could this plain English summary be used on its own to describe the proposed research? If not, what further information is needed?<br><br>Further information for researchers on how to write a plain English summary and what to include in a summary is available online at NIHR Make it clear http://www.invo.org.uk/makeitclear/.

Well written and clear, terms explained well

4. Strength of the research team

i) How well are the roles of the team members described? Is the overall team well coordinated?<br>ii) On the basis of track record in relevant areas, how qualified are the applicants to undertake the work using the methodologies proposed?<br>iii) To what extent does the research team have the necessary breadth and depth of expertise to deliver the proposed work (*e.g.* as judged by publication output and previous research funding)?<br>iv) How could the strength of the research team be improved?<br>v) If the lead applicant is inexperienced, does he/she have appropriate support (*e.g.* from their organisation and/or more senior colleagues) to deliver the work plan?
There seems a good plan in place for the team and to ensure oversight. There is an obvious depth of experience and track record in the field and in particular knowledge of NMA and Cochrane reviews. I am not aware if the team practice the techniques such as coeliac plexus block for technical assistance so having someone doing these procedures to discuss with would be useful. Also is there experience in e.g prehabiliation / nutrition from the cachexia side?<br><br>The ead applicant has appropriate support.

5. Impact of the proposed work

**Dissemination, outputs and anticipated impact**<br>NIHR aims to fund research that has the potential to be of significant benefit to the NHS, patients and the public. To support this, the applicants should consider how they will achieve impact from the outset. This helps them to identify potential impact and beneficiaries, and plan processes by which the research can directly, or incrementally over time, lead to change.<br><br>i) Have the applicants clearly expressed the problem and outlined how this research contributes towards a solution? Are the planned outputs appropriate and sufficient?<br>ii) Is it clear from the application who or which groups (including, as appropriate, healthcare planners, clinicians, patients and/or policy makers) are expected to benefit the most, how they will be engaged and communicated with, and if appropriate methods of engagement and dissemination are planned?<br>iii) Have the applicants set out appropriate activities and resources to achieve their impact goals? Is there a realistic trajectory and estimate of timescales for the benefit to reach patients/ public/ health and care services? Are there clear connections between outputs, engagement processes and impact goals?<br>iv) Have the applicants clearly considered what follow-on support they might need to generate or upscale impact, and how they might leverage further investment?<br>v) Have the applicants sufficiently and correctly identified any potential barriers they might face (e.g. IP, regulatory and acceptability) and have they properly considered how these may be overcome?<br>vi) Have the applicants clearly and realistically outlined the anticipated impacts, the likely scale of these impacts (both in the shorter and longer terms), and the sequence in which the impacts might occur?
1. Yes, we urgently need evidence based options for pain and cachexia in pancreatic cacer<br><br>2. Hopefully the work will find its way into guidelines and thus clinical practice, currently there is no real treatments validated and consistently used in the real world despite multiple rcts.<br><br>3. Yes and suitable public/patient engagement is written in. PCUK involved.<br><br>4. I don't think this is addressed.<br><br>5/6. Yes

6. Value for money

i) Taking into consideration the costs associated with undertaking the research, is there sufficient justification for the resources requested? Are all costs essential for the work proposed?<br>ii) Are appropriate resources set aside for patient and public involvement - including plans for a training and support budget?<br>iii) Where relevant, are resources to support impact - other than those for public and patient involvement - included and appropriate?<br>iv) Taking into account the total cost of the research, including the NHS costs, to what extent does the research provide value for money?<br>v) If required, are funds requested for NHS support and treatment costs appropriate and justified?
The costs are almost all in researcher time and seem appropriate.

7. Involvement of patients and the public

**Was there any patient and public involvement in the application?**	Yes

i) What is your assessment of the patient and public involvement (if any) in the development of the application, including involvement in: Identifying the research topic; prioritising the research questions; preparing the application (e.g. contributing to the research design); and identifying potential impact?<br>ii) What is your assessment of any proposed plans for patient and public involvement throughout the life of the research? Can you identify particular strengths, weaknesses and/or areas for improvement?
very well done. There is involvement of a carer, PCUK and focus groups.

8. Additional Comments

If you have any additional comments, not covered by the sections above, please provide them below.
